# The glycosylation-dependent interaction of perlecan core protein with LDL: implications for atherosclerosis[S]

**DOI:** 10.1194/jlr.M053017

**Published:** 2015-02

**Authors:** Yu-Xin Xu, David Ashline, Li Liu, Carlos Tassa, Stanley Y. Shaw, Katya Ravid, Matthew D. Layne, Vernon Reinhold, Phillips W. Robbins

**Affiliations:** * Center for Human Genetic Research and Cardiovascular Research CenterMassachusetts General Hospital, Boston, MA 02114; ** Center for Systems Biology, Massachusetts General Hospital, Boston, MA 02114; † The Glycomics Center, University of New Hampshire, Durham, NH 03824; § Department of Molecular and Cell Biology, Boston University Henry M. Goldman School of Dental Medicine, Boston, MA 02118; †† Departments of Medicine Boston University School of Medicine, Boston, MA 02118; §§ Biochemistry, Boston University School of Medicine, Boston, MA 02118

**Keywords:** low density lipoprotein receptor, sialic acid, low density lipoprotein

## Abstract

Perlecan is a major heparan sulfate (HS) proteoglycan in the arterial wall. Previous studies have linked it to atherosclerosis. Perlecan contains a core protein and three HS side chains. Its core protein has five domains (DI–DV) with disparate structures and DII is highly homologous to the ligand-binding portion of LDL receptor (LDLR). The functional significance of this domain has been unknown. Here, we show that perlecan DII interacts with LDL. Importantly, the interaction largely relies on *O*-linked glycans that are only present in the secreted DII. Among the five repeat units of DII, most of the glycosylation sites are from the second unit, which is highly divergent and rich in serine and threonine, but has no cysteine residues. Interestingly, most of the glycans are capped by the negatively charged sialic acids, which are critical for LDL binding. We further demonstrate an additive effect of HS and DII on LDL binding. Unlike LDLR, which directs LDL uptake through endocytosis, this study uncovers a novel feature of the perlecan LDLR-like DII in receptor-mediated lipoprotein retention, which depends on its glycosylation. Thus, perlecan glycosylation may play a role in the early LDL retention during the development of atherosclerosis.

CVD is, and will continue to be in the foreseeable future, the most common cause of death worldwide. In the United States, it kills more than 800,000 people annually (nearly one of every three deaths); the mortality is greater than any other disease (1). The leading cause of CVD is atherosclerosis, which is a pathological condition arising from fibrous plaque build-up inside the arterial wall. The plaque narrows the lumen of blood vessels and restricts blood flow. Rupture of the advanced plaque induces the formation of thrombus and blocks blood flow, which results in complications such as heart attack or stroke (2).

Elevated LDL level is a leading risk factor for atherosclerosis (3). The progressive accumulation of LDL in the vessel wall drives the development of atherosclerosis (4). The initiation of atherosclerosis may be mediated by the subendothelial retention of LDL (5, 6). This results from unbalanced dynamics of LDL, i.e., increased transfer to the arterial wall and retention by the extracellular matrix, mainly the proteoglycans (7, 8). For instance, in rabbit atherosclerosis models, injected LDL accumulated focally at atherosclerosis-prone regions of the arterial wall (9, 10). Similarly, transgenic mice expressing proteoglycan-binding defective LDLs exhibit a significantly lower rate of atherosclerosis compared with mice expressing WT LDL (11).

Proteoglycans are major components of the extracellular matrix lining the arterial wall (12, 13). Typically, proteoglycans consist of a core protein and one or multiple covalently linked glycosaminoglycans (GAGs) (14). The proteoglycan, perlecan, is normally synthesized by endothelial cells, before being deposited in the subendothelial extracellular matrix (15). Perlecan consists of a core protein with a molecular mass of ∼450 kDa and three long side chains of heparan sulfate (HS), which are critical in atherosclerosis development (16). The core protein has five domains (DI–DV) with disparate structural features. The N-terminal DI contains attachment sites for HS side chains. It is important to note that DII contains cysteine-rich repeat units that are highly homologous to the ligand-binding module of LDL receptor (LDLR).

Perlecan is abundantly present in the lesions of LDLR- or ApoE-deficient mice (17), and its expression correlates with lesion progression. In very advanced lesions with necrotic lipid cores, the level of perlecan was remarkably increased (18). Mice with a heterozygous deletion of perlecan exhibited a partially reduced expression of perlecan in the arterial wall and the deletion resulted in less atherosclerosis in young ApoE-deficient mice (18). Perlecan may contribute to atherosclerosis progression via its interaction with ApoB-100 (12, 16). The perlecan HS binds LDL and promotes LDL retention. Depletion of perlecan HS in ApoE-null mice significantly reduced atherosclerosis (16). Study of mouse perlecan indicates that DII forms a globular domain connected with a rod-like structure and is heavily glycosy­lated (19). However, the function of perlecan DII in LDL binding has never been investigated.

In this study, we demonstrate that the core protein of perlecan interacts with LDL via its LDLR-like DII. We found that the secreted DII was heavily modified with *O*-linked glycans and its interaction with LDL was largely dependent on the glycosylation. Interestingly, the glycans carry terminal sialic acid residues that are critical for the interaction. We found that both perlecan and its sialic acid modification are overexpressed in human atherosclerotic arterial wall. Collectively, our study provides new evidence that perlecan sialic acid-containing glycosylation might contribute to the development of atherosclerosis at the early stage.

## MATERIALS AND METHODS

### Cell culture and total cell extracts

HeLa, COS7, HEK293, and PAC-1 (rat vascular smooth muscle cells) cells were cultured in DMEM medium (Invitrogen) supplemented with 10% FBS (Sigma) and penicillin/streptomycin (Invitrogen). WT, ldlA, pgsa-745, Lec1, Lec2, and Lec8 Chinese hamster ovary (CHO) cells were cultured in Ham’s F-12 (Cellgro) with 10% FBS and penicillin/streptomycin. For secreted recombinant protein expression in media, transfected cells were grown in OPTI-MEM medium (Invitrogen) with 2% IgG-free FBS (Hyclone). Total cell extracts were prepared by solubilizing cells in a cell lysis buffer [0.5% Triton X-100, 0.5% sodium deoxycholate, 50 mM Tris-HCl (pH 7.5), 250 mM NaCl, 5 mM EDTA, and 50 mM sodium fluoride] in the presence of complete protease inhibitor cocktail (Roche).

### Oligonucleotide sequences, site-directed mutagenesis, and expression constructs

cDNAs for perlecan DII, DI, and DI+II were obtained by RT-PCR using the SuperScript first-strand synthesis system (Invitrogen). The DII, DI, and DI+II cDNAs were amplified by PCR (see supplemental information for primer sequences). Site-directed mutageneses were carried out by two-step PCR with various mutant primers carrying single or multiple base substitutions to change Ser/Thr to Gly/Ala and the forward and backward primers of DII. The cDNAs were subcloned into pcDNA3 plasmid at *Eco*RI/NotI sites. For the Fc-tagged (the Fc domain of human immunoglobulin protein) expression constructs, the Fc cDNA was amplified by PCR with an Fc-tagged plasmid as a template, a gift from Varki’s laboratory (20). The PCR product was subcloned into pcDNA3 at NotI/ApaI sites. A cDNA containing the secretion signal from BM40 was synthesized and subcloned into pcDNA3 at *Hin*dIII/*Eco*RI sites (19).

### Transfection and Western blotting

The expression constructs, as described above, were transfected into cells using Lipofectamine 2000 reagent or Lipofectamine LTX reagent (for PAC-1 cells) (Invitrogen) according to the manufacturer’s protocol. Western blotting was carried out with 10% SDS-PAGE, 4–15% gradient Mini-PROTEAN TGX precast gels (Bio-Rad), or 4–12% Bis-Tris precast gels (Invitrogen). Blots were probed with various antibodies, as indicated in the figures (see supplementary Materials and Methods for antibody information) with or without relevant HRP-labeled goat anti-mouse or goat anti-rabbit IgG (Sigma). For the lectin Western blotting, blots were blocked in blocking solution containing 50 mM Tris-HCl (pH 7.4), 0.15 M NaCl, 0.25% BSA, and 0.05% NP-40 at room temperature for 1 h, then incubated with various biotinylated lectins in the same buffer but with 1 mM MnCl_2_, 1 mM CaCl_2_, and 1 mM MgCl_2_ for 1 h. The lectins used in this study include *Griffonia simplicifolia* lectin (GSL) II, *Ulex europaeus* agglutinin (UEA) I, peanut agglutinin (PNA), wheat germ agglutinin (WGA), *Dolichos biflorus* agglutinin (DBA), concanavalin A, *Maackia amurensis* lectin (MAL) II, and *Sambucus nigra* agglutinin (SNA) (all were from Vector). After washing three times, blots were incubated with HRP-labeled streptavidin (Kirkegaard and Perry Lab) for 1 h. Following washing, blots were developed after treatment with ECL chemiluminescence reagent (GE Healthcare Life Sciences).

### Protein purification and in vitro pull-down assays

Recombinant proteins containing Fc-tagged perlecan DII, DI, DI+II, and Fc tag alone were purified from HEK293 cells, WT, or pgsa-745 mutant CHO cells. The Flag-tagged perlecan DII was purified from COS7 cells. For secreted recombinant proteins, culture medium from transfected cells was clarified by centrifugation. Cell extracts were prepared by solubilizing transfected cells in the above lysis buffer. The medium supernatant and cell extract were then incubated with protein-A agarose (RepliGen Corp.) or anti-Flag M2 affinity gel (Sigma) in TBS containing 50 mM Tris (pH 7.4), 150 mM NaCl, 0.05% Tween-20, and 1× protease inhibitor cocktail at 4°C overnight. The beads were then pelleted by centrifugation and washed three times with TBS and three times with the buffer containing 0.5 M NaCl. The bound proteins were eluted with 0.2 M glycine-HCl buffer (pH 3.0). All the purified proteins were dialyzed against a buffer containing 20 mM HEPES (pH 6.0) and 10 mM NaCl. Protein concentrations were measured by using Bio-Rad protein assay (Bio-Rad). Briefly, recombinant proteins were first incubated with protein-A agarose for Fc or Fc-tagged proteins in 1× PBS at 4°C overnight. The beads were precipitated by spin and washed with 1× PBS and the binding buffer, once each. The protein-bound beads were then incubated with human LDL (Biomedical Technologies, Inc.) or ApoB-100 (Sigma/Calbiochem) in a binding buffer containing 20 mM HEPES (pH 6.0), 10 mM NaCl, and 1% BSA or in TBS with 1% BSA (for supplementary Fig. 2) at room temperature for 1 h. The beads were precipitated by spin and washed three times with the binding buffer. The bound LDL/ApoB-100 was eluted with the glycine-HCl buffer and analyzed with 4–15% gradient precast gels.

### Surface plasmon resonance measurements

Surface plasmon resonance (SPR) measurements were performed on a Biacore T200 instrument (GE Healthcare) using Series S Sensor Chip C1 (matrix-free surface). Binding was measured at 25°C using the binding buffer, as described above, containing 0.2 mg/ml BSA. Binding data were analyzed using the evaluation software provided with the instrument. Briefly, the monoclonal anti-ApoB antibody was directly coupled to the sensor surface carboxyl groups using amine-coupling chemistry, as described before (21). Human ApoB-100 (50 μg/ml in PBS) was injected over the antibody surface for 420 s (10 μl/min), which resulted in ApoB-100 capture levels between 30 and 35 RU. A reference surface was prepared in the same manner without ApoB-100 capture. Flag-tagged perlecan DII was injected over both surfaces (60 s association phase, followed by 90 s dissociation phase at 30 μl/min) at increasing concentrations (1:2 dilution series from 0.063 to 1.0 μg/ml). The resulting binding response curves were double reference subtracted and globally fitted to a 1:1 binding model.

### Sugar mass spectrometry

The *O*-linked glycans of WT perlecan DII were released from proteins via slightly modified β-elimination using alkaline borohydride (22). The sample was desalted by passage through DOWEX 50W cation exchange resin (Sigma). Glycans were additionally purified by porous graphitized carbon (Agilent) solid phase extraction prior to permethylation. Permethylation was carried out in spin columns (Harvard Apparatus) as described (23). Purification of permethylated oligosaccharides was performed by liquid-liquid extraction with dichloromethane and 0.5 M aqueous sodium chloride. Qualitative analysis was performed via direct infusion via nanoelectrospray (Advion Nanomate, Ithaca, NY) into a Thermo LTQ (San Jose, CA) ion trap mass spectrometer.

### Immunohistochemistry and confocal microscopy

For cell confocal analysis, ldlA CHO cells were transfected with Fc or Fc-tagged perlecan DII in pcDNA3 plasmids. After 24 h, the cells were incubated with 1,1’-dioctadecyl-3,3,3’,3’-tetramethyl-indocarbocyanine perchlorate (Dil)-LDL (Biomedical Technologies, Inc.) as previously described (24). After wash, the cells were fixed with formaldehyde and stained with FITC-labeled anti-Fc antibody (Sigma) as described (25). For immunohistochemistry (IHC) analysis, human atherosclerosis and matched nonatherosclerosis frozen aorta sections (Biochain) were hybridized with the perlecan-specific rat monoclonal antibody (1:100, Millipore) or anti-ApoB mouse monoclonal antibody and the biotinylated MAL II lectin (1:200, Vector) in TBS buffer containing 5% normal donkey serum. After washing, the tissue sections were then incubated with Alexa 488-conjugated donkey anti-rat secondary antibody and Dylight 647-conjugated streptavidin (1:200, Jackson Immuno Research) in the same antibody dilution buffer. After rinsing in TBS, the tissue sections were mounted with Prolong Gold anti-fade mounting medium containing 4’,6-diamidino-2-phenylindole (DAPI) (Invitrogen). Confocal images were taken using a Zeiss LSM510 Meta confocal system with Zeiss LSM510 image acquisition software. The IHC and confocal microscopy assay were carried out in the Confocal Imaging Core facility at the Beth Israel Deaconess Medical Center.

## RESULTS

### Secreted perlecan DII strongly interacts with LDL

**Fig. 1.**
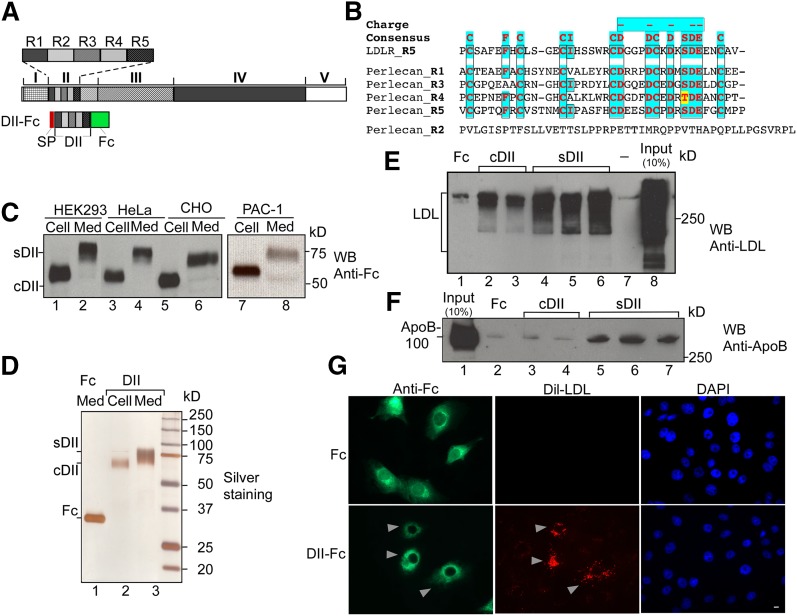
Perlecan DII strongly interacts with LDL in vitro. A: Schematic representation of perlecan DII structure and expression construct. DII has five repeat units (R1–R5). The construct for DII contains a C-terminal Fc tag and N-terminal secretion peptide (SP). B: Perlecan DII is highly homologous to the ligand-binding modules of LDLR. Sequence alignment of five repeat units of DII (Perlecan_R1–R5) with LDLR repeat 5 (LDLR_R5) was carried out with ClustalW2. The highly conserved residues are highlighted with colors. The consensus and negatively charged residues are shown on top. C: Expression analyses of perlecan DII. The DII expression construct, as in (A), was expressed in cells as indicated. Extracts from cells (Cell) and medium (Med) were analyzed with Western blotting (WB) and probed with an anti-Fc antibody. The cDII and sDII forms are indicated. D: Purification of perlecan recombinant DII. Silver staining of purified cDII and sDII, as well as Fc control, from a gradient SDS-PAGE. E: In vitro binding assay for the interaction of purified DII with human LDL. Equal amounts of the purified Fc, cDII, and sDII (500 ng) as in (D) or buffer only were used in the binding assay with human LDL (10 μg). The precipitated products were analyzed with Western blotting and detected with an antibody against LDL. The multiple lanes for cDII and sDII represent different preparations. F: A similar experiment, as in (E), was performed, but the blot was detected by an antibody against ApoB. G. Overexpression of perlecan DII enhances the LDL retention on the cell surface. The Fc tag (top) or Fc-tagged DII (bottom) plasmids were transfected into ldlA CHO cells. The transfected cells were incubated with Dil-LDL and then fixed and immunostained with FITC-labeled anti-Fc antibody. The cells with DII-Fc and Dil-LDL retention are indicated with arrows. Bar, 10 μM.

Perlecan DII has five sequence units designated R1–R5 (**Fig. 1A**) with four of them exhibiting homology to the ligand-binding modules of LDLR (15) (Fig. 1B). In spite of this homology, the binding activity of perlecan with LDL has never been examined. To test this concept, we generated a recombinant fragment of the perlecan DII with an Fc tag for purification and detection. Recombinant protein was expressed in HEK293, HeLa, CHO, and PAC-1 cells, and the protein present in the media [secreted DII (sDII)] exhibited a significantly slower mobility as compared with the cellular form [cellular DII (cDII)] (Fig. 1C), suggesting a posttranslational modification(s). The profile of purified cDII (lane 2), sDII (lane 3), and Fc (lane 1) is shown in Fig. 1D. To investigate the binding activity of DII with human LDL, we used in vitro pull-down assay and LDL-specific antibodies. The binding of sDII (Fig. 1E, lanes 4–6) with LDL was significantly greater than cDII (lanes 2 and 3). The binding with the Fc tag alone (lane 1) and buffer (lane 7) was used as a background control. A similar experiment was carried out, but detected with an ApoB-specific monoclonal antibody (Fig. 1F). ApoB-100 binding signal was only observed for sDII (lanes 5–7), but not for cDII (lanes 3 and 4). The results indicate that the perlecan DII strongly interacts with LDL and the interaction is largely dependent on the modification.

To validate whether the interaction actually occurs at the cell surface, we made use of ldlA CHO cells, which are LDLR deficient (24). We transfected Fc or Fc-tagged DII constructs into the ldlA cells. The transfected cells were then incubated with Dil-LDL and stained with FITC-labeled anti-Fc antibody. Overexpression of perlecan DII (bottom), but not the Fc tag alone (top), enhanced the retention of Dil-LDL at the cell surface (Fig. 1G), suggesting that the interaction indeed occurred at the cell surface under the physiological condition.

**Fig. 2.**
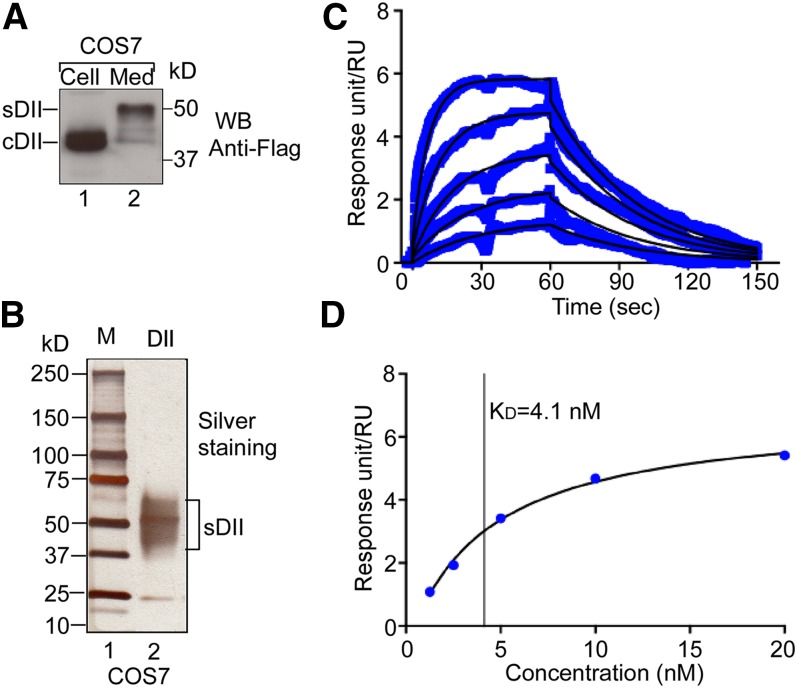
SPR analysis of the interaction between perlecan DII and ApoB-100. A: Expression of Flag-tagged DII. The Flag-tagged DII was expressed in COS7 cells. Extracts from cells (Cell) and medium (Med) were analyzed with Western blotting (WB) and probed with an HRP-conjugated anti-Flag antibody. The Flag-tagged sDII and cDII proteins are indicated. B: Purification of Flag-tagged sDII. The purified proteins were analyzed on 4–15% gradient SDS-PAGE with silver staining. C: SPR analyses of the interaction of perlecan DII with ApoB-100. Binding response (blue lines) was monitored in real-time (*t* = 0–60 s, association phase; *t* = 60–150 s, dissociation phase) for a series of increasing concentrations (1.3–20 nM in 1:2 dilution). Fitted curves modeled to describe a 1:1 binding event (overlaid in black) provide the association rate constant *k*_a_ (units of M^−1^s^−1^) and dissociation rate constant *k*_d_ (units of s^−1^). The equilibrium rate constant, *K*_D_, was calculated from the relation, *k*_d_/*k*_a_ = *K*_D_. D: Determination of the equilibrium rate constant, *K*_D_, from steady-state affinity data. Plot of steady-state binding as a function of analyte concentration fitted to a 1:1 steady-state binding model. The vertical line indicates the value of the calculated equilibrium rate constant, *K*_D_.

To quantify the binding affinity, we generated Flag-tagged DII and expressed it in COS7 cells. Consistent with the above results (Fig. 1C), the migration of sDII was slower than that of cDII (**Fig. 2A**, compare lane 2 with lane 1). The recombinant Flag-tagged sDII was purified (Fig. 2B) and then used in SPR assay for measuring its affinity and binding kinetics with ApoB-100 (Fig. 2C). We observed a high association rate (*k*_a_ = 5.9 × 10^6^ M^−1^s^−1^) and a relatively slow disassociation rate (*k*_d_ = 2.7 × 10^−2^ s^−1^). The high *k*_a_ value might reflect the fast binding between DII and ApoB-100. Interestingly, the high binding affinity at the optimal binding condition (*K*_D_ = 4.1 nM) (Fig. 2D) is comparable to that of LDLR with LDL (5–25 nM), as reported previously (26). To investigate the binding capacity of perlecan DII with LDL, we carried out an in vitro saturation binding analysis. The quantitative results (supplementary Fig. 1) indicate that the LDL binding to sDII (10.0 pM) became saturated when 16.3 pM LDL was used. Collectively, the above results indicate that the perlecan DII exhibits high affinity in LDL binding, which is largely dependent on its modification.

### The interaction of perlecan DII with LDL is dependent on *O*-glycans

**Fig. 3.**
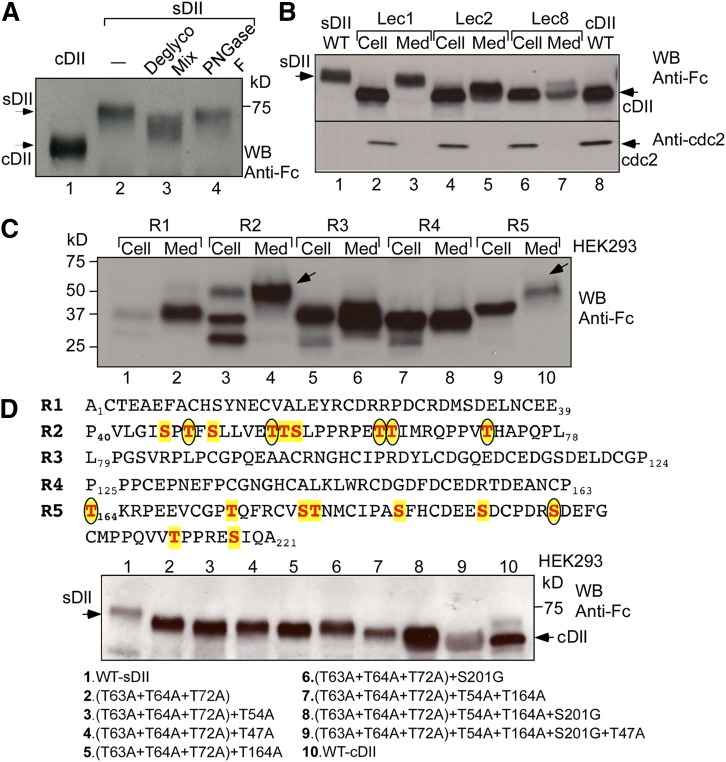
The secreted perlecan DII is glycosylated. A: Deglycosylation analysis of the secreted perlecan DII. Purified sDII, as in Fig. 1D, was incubated with a buffer only (lane 2), deglycosylation enzyme mix (Deglyco Mix) (lane 3), or PNGase F (lane 4). The products were analyzed with Western blotting (WB) along with cDII (lane 1) and probed with the anti-Fc antibody. B: Expression analyses of perlecan DII in WT and mutant CHO cell lines. The DII construct, as in Fig. 1A, was transfected into WT CHO (lanes 1 and 8), Lec 1 (lanes 2 and 3), Lec 2 (lanes 4 and 5), and Lec 8 (lanes 6 and 7). Extracts from cells (Cell) and medium (Med) were analyzed with Western blotting and detected by the anti-Fc antibody. sDII and cDII are indicated. C: Expression analyses of the individual DII repeats. Similar expression constructs for each repeat unit, as in Fig. 1A, were expressed in HEK293 cells and analyzed as in (B). The secreted recombinant repeats 2 and 5 with slower mobility are indicated with arrows. D: Mapping the glycosylation sites of perlecan DII. Site-directed mutageneses were generated by changing Ser/Thr to Gly/Ala. The WT and mutant DII constructs were expressed and analyzed as in (B). The mutants with multiple Ser/Thr mutations are listed at bottom. The Ser/Thr residues in repeats 2 and 5 are highlighted with color, and the confirmed glycosylation sites are marked with circles on top.

We next sought to determine the nature and functional importance of perlecan DII modifications. Deglycosylation of the purified Fc-tagged sDII with specific enzymes to remove both *O*- and *N*-linked glycans resulted in a significant mobility change (**Fig. 3A**, lane 3). These changes were not observed with peptide-N-glycosidase (PNGase) F (New England BioLabs) (lane 4), which specifically removes *N*-linked glycans. Inspection of the protein sequence did not reveal any typical *N*-linked glycosylation site (i.e., Asn-X-Ser/Thr, where X can be any other amino acids except proline), but it contained multiple Ser/Thr sites (Fig. 3D, top), suggesting that the modifications might be predominately *O*-linked glycosylation. The Fc-tagged DII was expressed in WT and several glycosylation-defective mutant CHO cell lines (Fig. 3B). The expression of sDII from Lec1 cells (lane 3), which are defective in *N*-linked glycosylation (27), was similar to that from WT CHO cells (lane 1), suggesting that DII indeed lacks *N*-linked glycans. The expression of DII in Lec2 cells (lanes 4 and 5), which are defective in sialic acid modification (28), and Lec8 cells (lanes 6 and 7), which are defective in both sialic acid and galactose modifications (29), indicate that it contains both sialic acid and galactose.

To further locate perlecan DII glycosylation sites, constructs containing each repeat unit were generated as the DII construct (Fig. 1A, B). The results in Fig. 3C show a dramatic mobility change for the secreted repeat 2 (compare lane 4 with lane 3), and a minor mobility change for the secreted repeat 5 (compare lane 10 with lane 9) as compared with their corresponding cellular forms, indicating that these repeats are glycosylated and the glycans from repeat 2 greatly contribute to the mobility of the secreted DII. In contrast, repeats 1, 3, and 4 have similar migration between the cellular and secreted forms (compare lane 1 with lane 2, lane 5 with lane 6, and lane 7 with lane 8, respectively), suggesting that these repeats are not modified. Notably, the second unit of DII is significantly deviated from a typical LDL-binding module and contains no cysteines, but is rich in Ser/Thr (Fig. 1B and Fig. 3D, top). In agreement with this observation, mouse perlecan has a similar sequence unit, which is also heavily glycosylated (19).

To map the glycosylation sites in repeats 2 and 5, we generated a series of 17 mutant constructs. After screening different combinations, we found that the mutant with combinations of threonine 63, 64, and 72 mutations from repeat 2 generated significant mobility change (Fig. 3D, bottom, lane 2), indicating that these sites are glycosy­lated. Therefore, based on this triple mutant, we added more single mutations for any additional mobility change. After screening various combinations, we found that addition of threonine 47 and 54 from repeat 2, and threonine 164 or serine 201 mutations from repeat 5 generated additional mobility changes (Fig. 3D, bottom, compare lanes 3–6 with lane 2). Sequential addition of these mutations resulted in mutant proteins with increasingly faster mobility (compare lanes 7 and 8 with lane 10), and the combination of all these mutations generated a mutant, whose secreted form is very similar to the WT cellular form (compare lane 9 with lane 10), suggesting that the mutant is defective in glycosylation. The confirmed glycosylation sites are circled in Fig. 3D (top), which are similar to the glycosylation positions in DII of mouse perlecan (19).

**Fig. 4.**
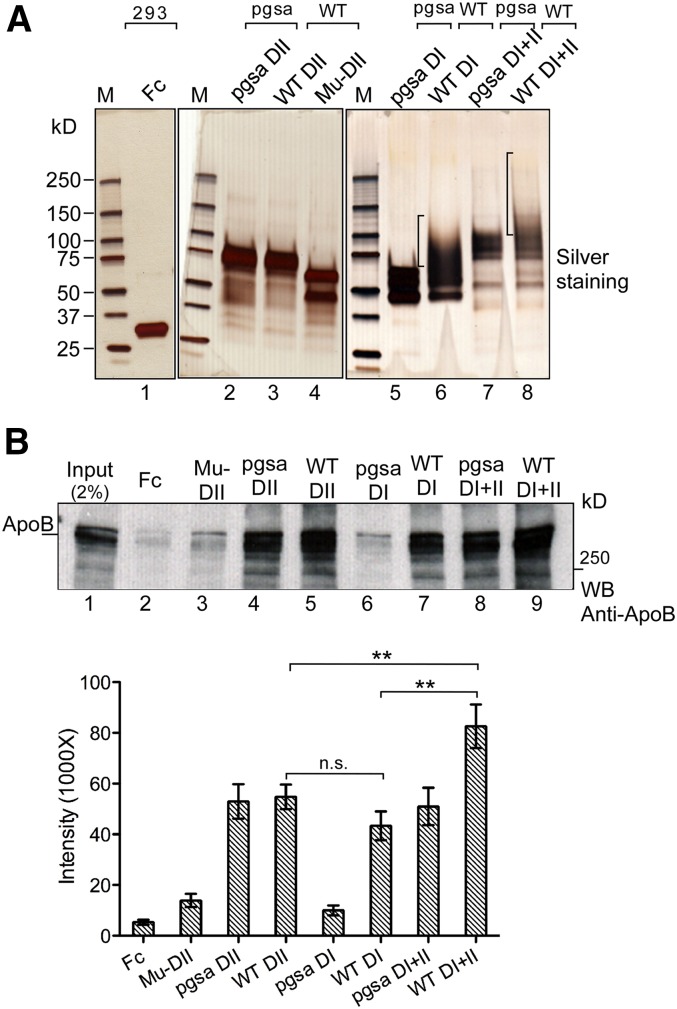
Functional analysis of perlecan DII and DI in ApoB-100 binding. A: Top, purification of perlecan DII, DI, and DI+II recombinant proteins. WT and Mu-DII, -DI, and -DI+II were purified from WT and pgsa-745 mutant CHO cells as indicated (Fc control from HEK293 cells). The glycosylation-defective Mu-DII was from the construct in Fig. 2D (lane 9). B: In vitro binding of the purified proteins with ApoB-100. Top, equal amounts of the purified proteins, as in (A), were used for in vitro binding assay as in Fig. 1E, except using human ApoB-100 (5 μg). The blot was probed with the anti-ApoB antibody. Bottom, quantitation of the in vitro binding assay. The average and standard error were based on three independent experiments. **P < 0.01 (*t*-test); n.s., *P* > 0.05.

To further confirm the glycosylation-dependent binding, we made use of the glycosylation-defective mutant DII (Fig. 3D, lane 9). We expressed and purified the secreted mutant (Mu)-DII from media along with the WT DII and Fc control (**Fig. 4A**, lanes 1–4). These proteins were used for the in vitro binding assays with human ApoB-100 protein. The results indicate that the mutations drastically reduced the binding of Mu-DII with ApoB-100, which is similar to the Fc control (Fig. 4B, top, compare lanes 3 and 2 with lanes 4 and 5), suggesting that the mutant is indeed inactive in ApoB binding.

### Coordinated activity of perlecan DII and DI in ApoB-100/LDL binding

The HS side chains on perlecan DI interact with LDL (16), and in this study we demonstrate that DII also interacts with LDL. To investigate whether there was any coordinated activity between DII and DI in LDL binding, we generated constructs that contained DI only and DI+II. The DI and DI+II, as well as WT DII constructs, were expressed in WT and mutant pgsa-745 (defective in glucuronosyltransferase I) CHO cells (30). The profile of the purified recombinant proteins (Fig. 4A) indicate that the DI (lane 6) and DI+II (lane 8) proteins from WT CHO cells were properly modified with HS, but not those from the mutant CHO cells (lanes 5 and 7, respectively), and the multiple bands of each protein likely reflect the heterologous sizes of HS side chains (see supplementary Fig. 2A). The DII proteins from both WT and mutant CHO cells have no obvious differences (lanes 3 and 2, respectively).

Equal amounts of the proteins were used for the in vitro binding assays with human ApoB-100 protein (Fig. 4B) under the optimal binding condition. The DII proteins from WT (lane 5) and mutant (lane 4) CHO cells show very similar binding activity, which is slightly higher than WT DI, but not statistically significant (24% increase, *P* > 0.05) (compare lanes 4 and 5 with lane 7). However, the binding of the DI+II from WT CHO cells is stronger than either WT DII or DI alone (53 and 90% increase, respectively, *P* < 0.01) (Fig. 4B, top; compare lane 9 with lanes 4-5, and lane 7; bottom for quantification). The binding of WT DI with ApoB was largely dependent on HS, because the lack of the modification of the DI from the pgsa-745 CHO cells greatly reduced the binding (compare lane 7 with lane 6). Similarly, the HS deficiency of the DI+II from the mutant CHO cells reduced its binding to the level of WT DII (compare lane 9 with lane 8). The results support the notion that there might be an additive effect between perlecan DII and DI on ApoB-100 binding. Similar results were obtained from the binding assay in TBS (supplementary Fig. 2). We also used a cell-based binding assay to further confirm the additive effect. We obtained stable cell lines expressing Fc, Fc-tagged DI, WT DII, DI+II, and Mu-DII from WT CHO cells. The stable cell lines were then used for binding with [I^125^]labeled LDL. The results show that expression of DI and WT DII increased the binding by ∼26 and ∼31%, respectively, whereas expression of DI+II increased about 43% (supplementary Fig. 3). The results are consistent with the finding from the in vitro ApoB-100 binding assay, suggesting that there might be a coordinated activity between perlecan HS on DII and DI in binding with LDL/ApoB-100.

### Sialylation in (α2-3) linkage of perlecan DII is critical for its LDL binding

**Fig. 5.**
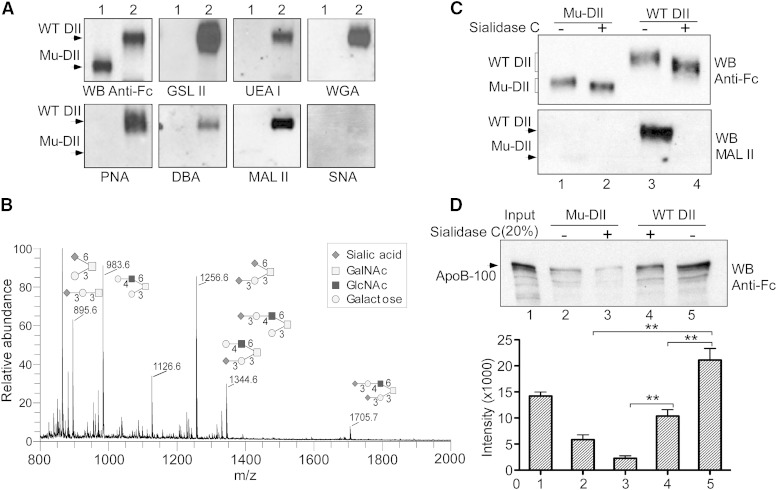
The structure and function analyses of perlecan DII *O*-linked glycans in LDL binding. A: Western blot analysis of WT and Mu-DII proteins with lectin probes. Equal amounts of the Fc-tagged Mu-DII and WT DII proteins, as in Fig. 4A, were analyzed on a gradient SDS-PAGE and probed with the anti-Fc antibody or the lectins, including GSL II (GlcNAc), UEA I (fucose), PNA (galactose), WGA (GlcNAc and sialic acid), DBA (GalNAc), MAL II, and SNA [(α2-3)- and (α2-6)-linked sialic acid, respectively]. B: Glycosylation profile of perlecan DII. *O*-linked glycans were released from purified Fc-tagged WT DII. The electrospray ionization-ion trap (ESI-IT) spectrum of *O*-linked glycans with possible isomers is shown. The symbols for each sugar are shown. C: Desialylation analysis of perlecan DII. The Fc-tagged WT DII and Mu-DII were treated with or without sialidase C and analyzed with Western blotting. Blots were probed with the Fc-specific antibody (top) or MAL II lectin (bottom). D: In vitro binding assay of desialylated DII in LDL binding. Top, Fc-tagged WT DII and Mu-DII treated with or without sialidase C, as in (C), were used for in vitro binding assay with human LDL (1.5 μg) as in Fig. 1E, and then analyzed with Western blotting. Blot was probed with the anti-ApoB antibody. Bottom, quantitation of the in vitro binding assay. The average and standard error were calculated from three independent experiments. **P < 0.01 (*t*-test).

The above results highlight the role of the *O*-linked glycans in mediating the interaction of perlecan DII with LDL. We next investigated the carbohydrate composition of the glycans using lectin Western blotting analysis. Equal amounts of the Fc-tagged WT and glycosylation-deficient mutant DII proteins were probed with the anti-Fc antibody or a series of biotinylated lectin probes. WT protein was recognized by the various lectins including GSL II for *N*-acetyl-glucosamine (GlcNAc), UEA I for fucose, PNA for galactose, WGA for GlcNAc, and sialic acid or DBA for *N*-acetylgalactosamine (GalNAc) (**Fig. 5A**). The results demonstrate that the glycans of DII likely contain GlcNAc, fucose, galactose, and GalNAc. Importantly, WT DII was also strongly recognized by MAL II, which is specific for sialic acid in an (α2-3) linkage, but not by SNA lectin, a lectin specific for sialic acid in an (α2-6) linkage (Fig. 5A, bottom right). None of the lectins tested recognized the mutant protein, confirming that it lacks the sugar modifications.

Next, we performed mass spectrometric analysis to define the sugar structure of the glycans. The most abundant five *O*-glycans with multiple possible isomers are presented in Fig. 5B. An interesting feature from these structures is that most of the glycans contain the terminal (α2-3)-linked sialic acids. This finding is consistent with the above expression analysis of DII in Lec2 and Lec8 cells (Fig. 3B) and the Western analysis with the sialic acid-specific lectins (Fig. 5A), indicating that (α2-3)-linked sialic acids significantly contribute to the *O*-linked glycans. Sialic acid linkages were determined empirically through sequential disassembly (supplementary Fig. 4) (31, 32).

To gain direct evidence that the sialylation plays a role in mediating the interaction of DII with LDL, we made use of sialidase C to remove the moieties from the purified proteins. Treatment with sialidase C significantly changed the mobility of the WT protein (Fig. 5C, top; compare lane 4 with lane 3). Consistently, the (α2-3)-linked sialic acid signal could not be detected by the MAL II lectin from the sialidase C-treated WT protein, confirming that the majority of the sialic acids were removed (Fig. 5C, bottom; compare lane 4 with lane 3). The sialidase C- or mock-treated WT and mutant proteins were purified and the protein-bound beads were then used for in vitro pull-down assay with human LDL. The precipitated LDL was analyzed with Western blotting detected with the anti-ApoB monoclonal antibody. Removal of sialic acid reduced the binding with LDL by ∼50% (Fig. 5D, top; compare lane 4 with lane 5 and bottom for quantification). Taken together, our results indicate that the perlecan DII contains significant (α2-3)-linked sialic acid modification, which augments the interaction of DII with LDL.

### Perlecan and its (α2-3)-linked sialic acid-containing glycans are overexpressed in human atherosclerotic lesions

**Fig. 6.**
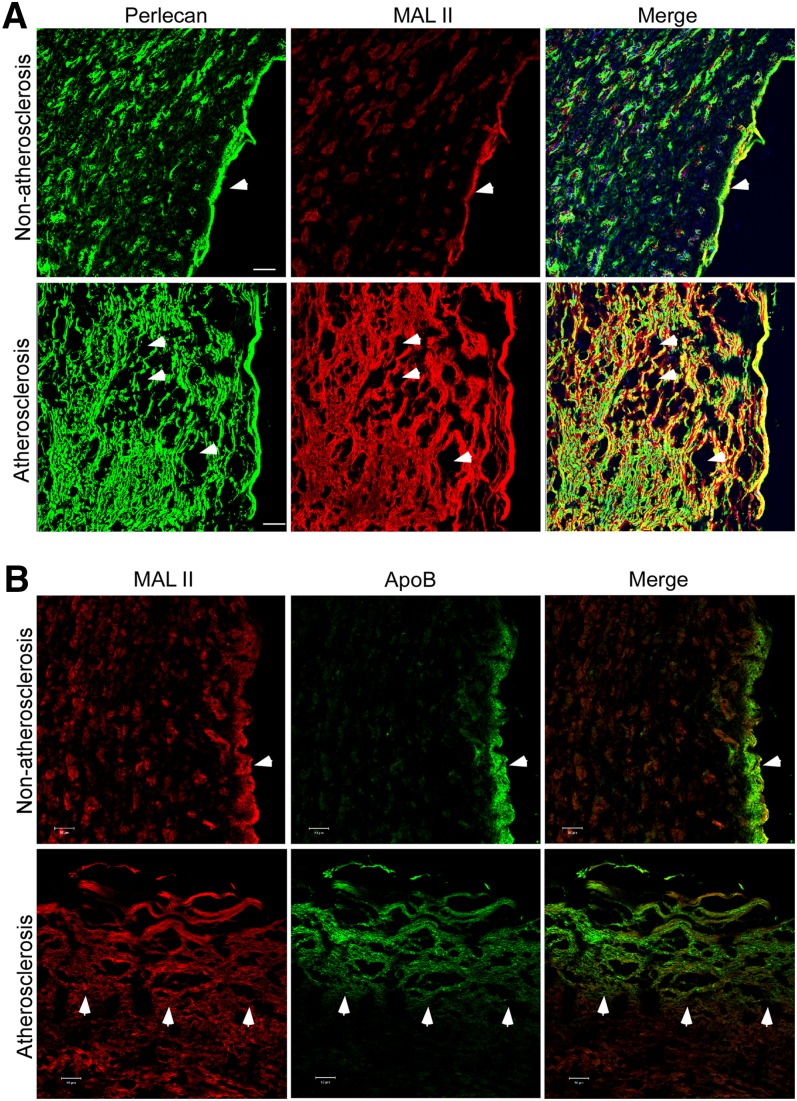
Perlecan and (α2-3)-linked sialic acid modification are overexpressed in human atherosclerotic arteries. Human normal and atherosclerotic arterial sections were stained with the perlecan-specific antibody (green) and MAL II lectin (red) (A) or the ApoB-specific monoclonal antibody (green) and MAL II lectin (red) (B), as indicated, and appropriate secondary antibodies. The images were obtained from a confocal microscope. The basement membrane in the normal tissue section (A and B, top), lipid accumulation area in the atherosclerotic section (A, bottom), and the edge of ApoB accumulation area (B, bottom) are indicated with arrows. Scale bar, 50 μm.

The finding from the sugar structure and function analyses prompted us to pursue an indirect clue for the sialic acid modification in the development of atherosclerosis. Human healthy and atherosclerotic arterial tissue sections were examined by IHC with a perlecan-specific monoclonal antibody and the biotinylated MAL II lectin. Perlecan and the (α2-3)-linked sialic acid were expressed at low levels across the healthy arterial wall with the most expression in the basement membrane (**Fig. 6A**, top). In comparison, in the atherosclerotic arterial section, perlecan was highly expressed in areas of lipid accumulation loci (empty area) in the arterial intima. The signal from (α2-3)-linked sialic acid modification was also elevated and colocalized with perlecan (Fig. 6A, bottom). The (α2-6)-linked sialic acid signal was barely detectable in both healthy and atherosclerotic arteries (data not shown).

To further investigate whether the sialic acid modification might be associated with the ApoB accumulation in the lesion, we stained the above tissue sections with the ApoB-specific monoclonal antibody and the MAL II lectin (Fig. 6B). We observed that, even in the healthy section, there was still significant ApoB signal at the area close to the luminal side of arteries (top panel), which may represent the entry site of LDL from blood. The ApoB accumulation was colocalized with the (α2-3)-linked sialic acid signal at the region rich in proteoglycans. In the atherosclerotic section, the ApoB signal was much more expanded into the arterial intima, and its distribution pattern was consistent with the MAL II staining (bottom panel), indicating that the (α2-3)-linked sialic acid modification was colocalized with ApoB accumulation. Taken together, these data suggest that perlecan and its sialic acid modification might be involved in the development of atherosclerosis.

## DISCUSSION

Previous studies have linked perlecan with atherosclerosis. Perlecan is overexpressed in the atherosclerotic lesions and its expression correlates with lesion progression. It was proved that the role of perlecan in atherosclerosis is based on the ionic interaction between the negatively charged HS on DI and the positively charged domains of ApoB-100 (16). Perlecan contains LDLR-like DII. Costell et al. (19) reported that the domain contains multiple *O*-linked glycosylation sites. However, the function of perlecan DII and its glycosylation in LDL binding has never been investigated. In this study, we demonstrate that the core protein interacts with LDL via its LDLR-like DII and the interaction is largely dependent on the *O*-linked glycans only present in the secreted DII. We found that most of the glycans are capped with the negatively charged sialic acids in (α2-3) linkage, which are important for the glycans’ function in LDL binding. We further determined that the expression of perlecan and sialic acid modification is elevated in the human atherosclerotic lesions, indicating that the *O*-linked glycans might be associated with the development of atherosclerosis.

The finding that the perlecan DII interacts with LDL suggests that the core protein may play a role in the LDL retention in the arterial wall that was not previously appreciated. The elevated expression makes it possible for the arterial perlecan (Fig. 6A) to interact with the LDLs that penetrate through the endothelial cells from the bloodstream and the interaction may facilitate the subendothelial LDL retention. It has been well-documented that the subendothelial retention of LDL is a critical step in the development of atherosclerosis (6). For example, using step and serial sections of coronary arteries, it was clearly shown that the earliest stage of atherosclerosis appears in a form of fatty streak that arises from the deposition of ApoB-containing lipoproteins (5). The event occurs much earlier than the filtration of macrophages in the early lesion. Various studies have validated that the proteoglycans, including perlecan and biglycan as well as versican, interact with LDL (16, 33, 34). Common to all the proteoglycans is that they contain sulfated GAGs. The interaction between the negatively charged sulfated GAGs and the positively charged domains of ApoB was proposed to be the molecular basis for the lipoprotein retention. The functionality of the perlecan LDLR-like DII characterized from this study indicates a glycosylation-dependent LDL binding as compared with the ionic interaction between the GAG and LDL. The perlecan domain may represent a distinct type of LDLR-like family. However, perlecan contains only the LDLR-like ligand-binding domain. It lacks the elements that are essential for the LDL internalization and endocytosis as a typical LDLR (35). Thus, the binding of the domain with LDL and its cooperative effect with HS on LDL binding might facilitate the LDL subendothelial retention in the arterial wall.

Perlecan DII possesses some unique structural features that likely mediate LDL binding. Among its five sequence units, four are perfectly homologous to the LDL binding modules of LDLR, and these conserved repeat units possess all the elements essential for ligand binding (Fig. 1B). For example, each of the repeats has six cysteines to form three disulfide bonds, and a cluster of negatively charged amino acids (DCXDXSDE, X represents any amino acids) near to the C terminus (Fig. 1B), which are complementary to the positively charged sequence of ApoB-100. The prototype LDLR contains seven such repeats, and the detailed structural and functional analyses demonstrate that repeats 3–7 are required for the LDL binding, and deletion of any of the five repeats dramatically reduces the binding activity (36). The second unit of perlecan DII is highly divergent. There are no conserved cysteine and negatively charged amino acids. Moreover, this repeat disrupts the consecutive stretch of the perfectly conserved repeats. The presence of such a repeat unit would make DII unlikely to be functional in LDL binding. Indeed, the cellular DII and the glycosylation-deficient mutant have a very weak activity in LDL binding. We found that the secreted domain strongly interacts with LDL in a glycosylation-dependent manner. Interestingly, most of the glycosylation sites of DII come from the second unit that is rich in Ser/Thr. A possible explanation for the glycosylation is that most of the *O*-linked glycans are capped with terminal sialic acids, which are known to be negatively charged (37). The finding was further confirmed by the removal of the sialic acids, which decreased the binding. This observation is consistent with the critical role of the cluster of negatively charged amino acids of LDLR in LDL binding. Mutations of the amino acids greatly reduced the LDL binding activity. The central role of the conserved acidic residues is also reflected in the crystal structure of the LDLR repeat units (38). The residues are clustered on the surface of the repeat that enables electrostatic contacts with the basic residues on the surface of ApoB-100. Thus the results from this study suggest that the sialic acid-containing glycans might be able to surrogate the requirement of acidic amino acids. However, the detailed structure for this remains to be investigated.

Our data suggest that the (α2-3)-linked sialic acid, but not (α2-6)-linked sialic acid, is involved in the perlecan-LDL interaction. The preference may be linked to the tissue- or protein-specific sialic acid linkage distribution. Study of avian and human influenza virus receptors, (α2-3)- and (α2-6)-linked sialic acids, respectively, showed that they are extensively presented in all organs in the pig, but with a distinct spatial distribution pattern (39, 40). In this case, perlecan DII is specifically modified with the sialic acid in (α2-3) linkage (Fig. 5). However, it remains to be determined whether there is any sialic acid linkage specificity for the perlecan-LDL interaction. The perlecan-LDL interaction possesses some unusual biochemical properties. The most significant one is that the interaction prefers acidic pH, which is different from the LDL-LDLR interaction. As described above, perlecan is an extracellular matrix protein. Characteristic to the extracellular macromolecules is their acidic nature because of the abundance of acidic amino acids, often with a high portion of aspartic acid (41). In addition, as for perlecan DII, the property may also be related to sialylation. Consistent with this finding, it was previously reported that the interaction of human aortic proteoglycans with LDL is strongly enhanced by acidic pH (42).

Our finding of the lesional overexpression of perlecan and its sialic acid-glycans is consistent with the notion that focal overexpression of proteoglycans may accelerate the progression of atherosclerosis (6). Perlecan was shown to be overexpressed in the early lesion both in ApoE(−/−) and LDLR(−/−) mouse models and its expression levels correlate with the atherogenic progression (17, 18). Our study shows that it is also overexpressed in human atherosclerotic arteries. The overexpressed perlecan is likely synthesized from the filtrated monocyte or monocyte-derived macrophages and/or smooth muscle cells at the lesional site (43). It is well-documented that oxidized LDL is able to stimulate endothelial cells to express inflammatory stimuli that recruit monocytes and smooth muscle cells to the arterial wall (44). The oxidized LDL itself, especially the extensively oxidized LDL, is chemotactic to these cells (45, 46). However, it was found that the monocyte-derived macrophages secreted the proteoglycans primarily containing chondroitin sulfate/dermatan sulfate GAGs, but only a minor amount of HS GAGs (47). Therefore, we presume that the overexpressed perlecan is most likely from the smooth muscle cells. It was shown that the proteoglycans synthesized from the established lesions retain more atherogenic lipoproteins. This study demonstrates that perlecan overexpression is accompanied with the heavy modification of sialic acid-containing *O*-linked glycans, which in turn enhance the interaction with LDL.

In summary, our data provide evidence that the perlecan core protein interacts with LDL. We show that the perlecan-LDL interaction is mediated by its LDLR-like DII in a manner dependent on sialic acid-containing *O*-glycans. Thus, the perlecan sialic acid modification is associated with the development of atherosclerosis and further study is needed to validate this conclusion.

## Supplementary Material

Supplemental Data

## References

[1] Go A. S. Mozaffarian D. Roger V. L. Benjamin E. J. Berry J. D. Blaha M. J. Dai S. Ford E. S. Fox C. S. Franco S. 2014 Heart disease and stroke statistics–2014 update: a report from the American Heart Association. Circulation. 129: e28–e292. 24352519 10.1161/01.cir.0000441139.02102.80 PMC5408159

[2] Libby P. Ridker P. M. Hansson G. K. 2011 Progress and challenges in translating the biology of atherosclerosis. Nature. 473: 317–325. 21593864 10.1038/nature10146

[3] Tsimikas S. Witztum J. L. 2008 The role of oxidized phospholipids in mediating lipoprotein(a) atherogenicity. Curr. Opin. Lipidol. 19: 369–377. 18607184 10.1097/MOL.0b013e328308b622

[4] Williams K. J. Tabas I. 1995 The response-to-retention hypothesis of early atherogenesis. Arterioscler. Thromb. Vasc. Biol. 15: 551–561. 7749869 10.1161/01.atv.15.5.551 PMC2924812

[5] Nakashima Y. Fujii H. Sumiyoshi S. Wight T. N. Sueishi K. 2007 Early human atherosclerosis: accumulation of lipid and proteoglycans in intimal thickenings followed by macrophage infiltration. Arterioscler. Thromb. Vasc. Biol. 27: 1159–1165. 17303781 10.1161/ATVBAHA.106.134080

[6] Tabas I. Williams K. J. Boren J. 2007 Subendothelial lipoprotein retention as the initiating process in atherosclerosis: update and therapeutic implications. Circulation. 116: 1832–1844. 17938300 10.1161/CIRCULATIONAHA.106.676890

[7] Proctor S. D. Vine D. F. Mamo J. C. 2002 Arterial retention of apolipoprotein B(48)- and B(100)-containing lipoproteins in atherogenesis. Curr. Opin. Lipidol. 13: 461–470. 12352009 10.1097/00041433-200210000-00001

[8] Nordestgaard B. G. Nielsen L. B. 1994 Atherosclerosis and arterial influx of lipoproteins. Curr. Opin. Lipidol. 5: 252–257. 7981955 10.1097/00041433-199408000-00002

[9] Nievelstein P. F. Fogelman A. M. Mottino G. Frank J. S. 1991 Lipid accumulation in rabbit aortic intima 2 hours after bolus infusion of low density lipoprotein. A deep-etch and immunolocalization study of ultrarapidly frozen tissue. Arterioscler. Thromb. 11: 1795–1805. 1931881 10.1161/01.atv.11.6.1795

[10] Schwenke D. C. Carew T. E. 1989 Initiation of atherosclerotic lesions in cholesterol-fed rabbits. I. Focal increases in arterial LDL concentration precede development of fatty streak lesions. Arteriosclerosis. 9: 895–907. 2590067 10.1161/01.atv.9.6.895

[11] Skålén K. Gustafsson M. Rydberg E. K. Hulten L. M. Wiklund O. Innerarity T. L. Boren J. 2002 Subendothelial retention of atherogenic lipoproteins in early atherosclerosis. Nature. 417: 750–754. 12066187 10.1038/nature00804

[12] Camejo G. Hurt-Camejo E. Wiklund O. Bondjers G. 1998 Association of apo B lipoproteins with arterial proteoglycans: pathological significance and molecular basis. Atherosclerosis. 139: 205–222. 9712326 10.1016/s0021-9150(98)00107-5

[13] Tannock L. R. King V. L. 2008 Proteoglycan mediated lipoprotein retention: a mechanism of diabetic atherosclerosis. Rev. Endocr. Metab. Disord. 9: 289–300. 18584330 10.1007/s11154-008-9078-0

[14] Varki A. Cummings R. Esko J. Freeze H. Hart G. Marth J. 1999. Essentials of Glycobiology. Cold Spring Harbor Press, Cold Spring Harbor, New York. 20301239

[15] Iozzo R. V. 2005 Basement membrane proteoglycans: from cellar to ceiling. Nat. Rev. Mol. Cell Biol. 6: 646–656. 16064139 10.1038/nrm1702

[16] Tran-Lundmark K. Tran P. K. Paulsson-Berne G. Friden V. Soininen R. Tryggvason K. Wight T. N. Kinsella M. G. Boren J. Hedin U. 2008 Heparan sulfate in perlecan promotes mouse atherosclerosis: roles in lipid permeability, lipid retention, and smooth muscle cell proliferation. Circ. Res. 103: 43–52. 18596265 10.1161/CIRCRESAHA.108.172833 PMC2765377

[17] Kunjathoor V. V. Chiu D. S. O’Brien K. D. LeBoeuf R. C. 2002 Accumulation of biglycan and perlecan, but not versican, in lesions of murine models of atherosclerosis. Arterioscler. Thromb. Vasc. Biol. 22: 462–468. 11884291 10.1161/hq0302.105378

[18] Vikramadithyan R. K. Kako Y. Chen G. Hu Y. Arikawa-Hirasawa E. Yamada Y. Goldberg I. J. 2004 Atherosclerosis in perlecan heterozygous mice. J. Lipid Res. 45: 1806–1812. 15258195 10.1194/jlr.M400019-JLR200

[19] Costell M. Sasaki T. Mann K. Yamada Y. Timpl R. 1996 Structural characterization of recombinant domain II of the basement membrane proteoglycan perlecan. FEBS Lett. 396: 127–131. 8914972 10.1016/0014-5793(96)01082-4

[20] Angata T. Varki A. 2000 Cloning, characterization, and phylogenetic analysis of siglec-9, a new member of the CD33-related group of siglecs. Evidence for co-evolution with sialic acid synthesis pathways. J. Biol. Chem. 275: 22127–22135. 10801860 10.1074/jbc.M002775200

[21] Tassa C. Liong M. Hilderbrand S. Sandler J. E. Reiner T. Keliher E. J. Weissleder R. Shaw S. Y. 2012 On-chip bioorthogonal chemistry enables immobilization of in situ modified nanoparticles and small molecules for label-free monitoring of protein binding and reaction kinetics. Lab Chip. 12: 3103–3110. 22760641 10.1039/c2lc40337d PMC3411869

[22] Carlson D. M. 1966 Oligosaccharides isolated from pig submaxillary mucin. J. Biol. Chem. 241: 2984–2986. 5912370

[23] Kang P. Mechref Y. Klouckova I. Novotny M. V. 2005 Solid-phase permethylation of glycans for mass spectrometric analysis. Rapid Commun. Mass Spectrom. 19: 3421–3428. 16252310 10.1002/rcm.2210 PMC1470644

[24] Acton S. Rigotti A. Landschulz K. T. Xu S. Hobbs H. H. Krieger M. 1996 Identification of scavenger receptor SR-BI as a high density lipoprotein receptor. Science. 271: 518–520. 8560269 10.1126/science.271.5248.518

[25] Xu Y. X. Liu L. Caffaro C. E. Hirschberg C. B. 2010 Inhibition of Golgi apparatus glycosylation causes endoplasmic reticulum stress and decreased protein synthesis. J. Biol. Chem. 285: 24600–24608. 20529871 10.1074/jbc.M110.134544 PMC2915696

[26] Fisher T. S. Lo Surdo P. Pandit S. Mattu M. Santoro J. C. Wisniewski D. Cummings R. T. Calzetta A. Cubbon R. M. Fischer P. A. 2007 Effects of pH and low density lipoprotein (LDL) on PCSK9-dependent LDL receptor regulation. J. Biol. Chem. 282: 20502–20512. 17493938 10.1074/jbc.M701634200

[27] Puthalakath H. Burke J. Gleeson P. A. 1996 Glycosylation defect in Lec1 Chinese hamster ovary mutant is due to a point mutation in N-acetylglucosaminyltransferase I gene. J. Biol. Chem. 271: 27818–27822. 8910379 10.1074/jbc.271.44.27818

[28] Eckhardt M. Gotza B. Gerardy-Schahn R. 1998 Mutants of the CMP-sialic acid transporter causing the Lec2 phenotype. J. Biol. Chem. 273: 20189–20195. 9685366 10.1074/jbc.273.32.20189

[29] Oelmann S. Stanley P. Gerardy-Schahn R. 2001 Point mutations identified in Lec8 Chinese hamster ovary glycosylation mutants that inactivate both the UDP-galactose and CMP-sialic acid transporters. J. Biol. Chem. 276: 26291–26300. 11319223 10.1074/jbc.M011124200

[30] Bai X. Wei G. Sinha A. Esko J. D. 1999 Chinese hamster ovary cell mutants defective in glycosaminoglycan assembly and glucuronosyltransferase I. J. Biol. Chem. 274: 13017–13024. 10224052 10.1074/jbc.274.19.13017

[31] Anthony R. M. Nimmerjahn F. Ashline D. J. Reinhold V. N. Paulson J. C. Ravetch J. V. 2008 Recapitulation of IVIG anti-inflammatory activity with a recombinant IgG Fc. Science. 320: 373–376. 18420934 10.1126/science.1154315 PMC2409116

[32] Ashline D. J. Hanneman A. J. Zhang H. Reinhold V. N. 2014 Structural documentation of glycan epitopes: sequential mass spectrometry and spectral matching. J. Am. Soc. Mass Spectrom. 25: 444–453. 24385394 10.1007/s13361-013-0776-9 PMC3950938

[33] Flood C. Gustafsson M. Richardson P. E. Harvey S. C. Segrest J. P. Boren J. 2002 Identification of the proteoglycan binding site in apolipoprotein B48. J. Biol. Chem. 277: 32228–32233. 12070165 10.1074/jbc.M204053200

[34] Llorente-Cortés V. Otero-Vinas M. Hurt-Camejo E. Martinez-Gonzalez J. Badimon L. 2002 Human coronary smooth muscle cells internalize versican-modified LDL through LDL receptor-related protein and LDL receptors. Arterioscler. Thromb. Vasc. Biol. 22: 387–393. 11884279 10.1161/hq0302.105367

[35] Rudenko G. Henry L. Henderson K. Ichtchenko K. Brown M. S. Goldstein J. L. Deisenhofer J. 2002 Structure of the LDL receptor extracellular domain at endosomal pH. Science. 298: 2353–2358. 12459547 10.1126/science.1078124

[36] Russell D. W. Brown M. S. Goldstein J. L. 1989 Different combinations of cysteine-rich repeats mediate binding of low density lipoprotein receptor to two different proteins. J. Biol. Chem. 264: 21682–21688. 2600087

[37] Varki A. 2007 Glycan-based interactions involving vertebrate sialic-acid-recognizing proteins. Nature. 446: 1023–1029. 17460663 10.1038/nature05816

[38] Jeon H. Blacklow S. C. 2005 Structure and physiologic function of the low-density lipoprotein receptor. Annu. Rev. Biochem. 74: 535–562. 15952897 10.1146/annurev.biochem.74.082803.133354

[39] Trebbien R. Larsen L. E. Viuff B. M. 2011 Distribution of sialic acid receptors and influenza A virus of avian and swine origin in experimentally infected pigs. Virol. J. 8: 434. 21902821 10.1186/1743-422X-8-434 PMC3177912

[40] Nelli R. K. Kuchipudi S. V. White G. A. Perez B. B. Dunham S. P. Chang K. C. 2010 Comparative distribution of human and avian type sialic acid influenza receptors in the pig. BMC Vet. Res. 6: 4. 20105300 10.1186/1746-6148-6-4 PMC2832630

[41] Weiner S. Addadi L. 1991 Acidic macromolecules of mineralized tissues: the controllers of crystal formation. Trends Biochem. Sci. 16: 252–256. 1926334 10.1016/0968-0004(91)90098-g

[42] Sneck M. Kovanen P. T. Oorni K. 2005 Decrease in pH strongly enhances binding of native, proteolyzed, lipolyzed, and oxidized low density lipoprotein particles to human aortic proteoglycans. J. Biol. Chem. 280: 37449–37454. 16147996 10.1074/jbc.M508565200

[43] Camejo G. Fager G. Rosengren B. Hurt-Camejo E. Bondjers G. 1993 Binding of low density lipoproteins by proteoglycans synthesized by proliferating and quiescent human arterial smooth muscle cells. J. Biol. Chem. 268: 14131–14137. 8314779

[44] Cushing S. D. Berliner J. A. Valente A. J. Territo M. C. Navab M. Parhami F. Gerrity R. Schwartz C. J. Fogelman A. M. 1990 Minimally modified low density lipoprotein induces monocyte chemotactic protein 1 in human endothelial cells and smooth muscle cells. Proc. Natl. Acad. Sci. USA. 87: 5134–5138. 1695010 10.1073/pnas.87.13.5134 PMC54276

[45] Quinn M. T. Parthasarathy S. Fong L. G. Steinberg D. 1987 Oxidatively modified low density lipoproteins: a potential role in recruitment and retention of monocyte/macrophages during atherogenesis. Proc. Natl. Acad. Sci. USA. 84: 2995–2998. 3472245 10.1073/pnas.84.9.2995 PMC304787

[46] Autio I. Jaakkola O. Solakivi T. Nikkari T. 1990 Oxidized low-density lipoprotein is chemotactic for arterial smooth muscle cells in culture. FEBS Lett. 277: 247–249. 2269361 10.1016/0014-5793(90)80857-f

[47] Khalil M. F. Wagner W. D. Goldberg I. J. 2004 Molecular interactions leading to lipoprotein retention and the initiation of atherosclerosis. Arterioscler. Thromb. Vasc. Biol. 24: 2211–2218. 15472124 10.1161/01.ATV.0000147163.54024.70

