# Supplementary material for: The glycosylation-dependent interaction of perlecan core protein with LDL: implications for atherosclerosis
Source: J Lipid Res. 2015 Feb;56(2):266–76. doi: 10.1194/jlr.M053017 (PMC4306681; doi:10.1194/jlr.M053017)
Supplement: Supplemental Data [file supp_56_2_266__index.html]

The glycosylation-dependent interaction of perlecan core protein with LDL: implications for atherosclerosis — The glycosylation-dependent interaction of perlecan core protein with LDL: implications for atherosclerosis — Supplemental Data 

# The glycosylation-dependent interaction of perlecan core protein with LDL: implications for atherosclerosis

## Supplemental Data

**Files in this Data Supplement:**

- Supplemental Information - Supplemental information includes materials and methods, figures and legends, and references.
