# Supplementary material for: The glycosylation-dependent interaction of perlecan core protein with LDL: implications for atherosclerosis
Source: J Lipid Res. 2015 Feb;56(2):266–76. doi: 10.1194/jlr.M053017 (PMC4306681; doi:10.1194/jlr.M053017)
Supplement: Supplemental Data [file supp_M053017_jlr.M053017-1.pdf]

Supplemental materials and methods

Antibodies, lectins, ApoB/lipoproteins, and frozen arterial sections

Anti-human IgG (Fc specific)-FITC antibody (goat) catalogue # F9512, Sigma

Anti-human IgG (Fc specific)-peroxidase antibody (goat) catalogue # A0170, Sigma

Monoclonal anti-Flag M2-peroxidase antibody (mouse) catalogue # A8592, Sigma

Monoclonal anti-ApoB antibody, catalogue # sc-13538, Santa Cruz Biotechnology,

Anti-cdc2 antibody, catalogue # sc-954, (rabbit), Santa Cruz Biotechnology

Anti-perlecan-specific monoclonal antibody, clone A7L6, catalogue # MAB1948P, Millipore, CA, USA

Alexa 488-conjugated anti-rat secondary antibody, catalogue # 712-545-153, Jackson Immuno Research

Alexa 647-conjugated donkey anti-mouse secondary antibody, catalogue # 715-605-161, Jackson Immuno Research

Alexa 647-conjugated streptavidin, catalogue # 016-600-084, Jackson Immuno Research

Human plasma Dil-LDL, catalogue # BT-904, Biomedical Technologies, Inc., MA, USA

Anti-human LDL antibody (rabbit), catalogue # BT-905, Biomedical Technologies, Inc., MA, USA

Human plasma LDL, catalogue # BT-903, Biomedical Technologies, Inc., MA, USA

Human plasma ApoB-100, cat#5353, Sigma

Human plasma ApoB-100, catalogue # 178456, Millipore

Biotinylated *Griffonia simplicifolia* lectin (GSL) II, catalogue # B-1215, Vector Laboratories, CA, USA

Biotinylated *Ulex Europaeus* agglutinin (UEA) I, catalogue # B-1065, Vector Laboratories

Biotinylated Peanut Agglutinin (PNA), catalogue # B-1075, Vector Laboratories

Biotinylated wheat germ agglutinin (WGA), catalogue # B-1025, Vector Laboratories

Biotinylated *Dolichos biflorus* agglutinin (DBA), catalogue # B-1035, Vector Laboratories

Biotinylated Concanavalin A (Con A), catalogue # B-1005, Vector Laboratories

Biotinylated *Maackia Amurensis* Lectin (MAL) II, catalogue # B-1265, Vector Laboratories

Biotinylated *Sambucus Nigra* (SNA), catalogue # B-1305, Vector Laboratories

Peroxidase-labeled streptavidin, catalogue # 14-30-00, Kirkegaard & Perry Laboratories.

Immobilized rProtein-A agarose, catalogue # 10-2500-02, RepliGen Corporation, MA USA

Frozen Tissue Section human arteriosclerosis Aorta and matched Non- arteriosclerosis aorta, catalogue # T6236012Hd-4-PP, BioChain

Primer sequences and mutant oligonucleotides

Forward primer for perlecan domain II cDNA: 5'-GCCTGCACGGAGGCCGAGT-3', and backward primer 5'-AGCCTGGATGGACTCCCGG-3'.

Forward primer for perlecan domain I cDNA: 5'-GTGACCCATGGGCTGAGG-3', and backward primer 5'-TCTTGGGAACTGGGGCACTG-3'.

Forward primer for Fc cDNA: 5'-GAGCCCAAATCTTGTGACAAAAC-3', and backward primer 5'-CTTGTCATCGTCATCCTTGTA-3'

Mutant oligonucleotides for perlecan DII mutagenesis (point base mutation is underlined)

S45G mutation, 5'-CCAGTCCTGGGTATCGGCCCCACATTCTCTC-3'

T47A mutation, 5'-ATC*A*GCCCCACATTCGCTCTCCTTGTGGAGA-3'

T54A mutation, 5’-TCTCTCCTTGTGGAGGCG*A*CATCTTTACCGCCCC-3’

T55A mutation, 5’-TCTCTCCTTGTGGAG*A*CGGCATCTTTACCGCCCC-3’

T54A+T55A mutations, 5’-TCTCTCCTTGTGGAGGCGGCATCTTTACCGCCCC-3’

S56A mutation, 5’-CTTGTGGAGACGACAGCTTTACCGCCCCGGC-3’

T63A mutation, 5’-CCGCCCCGGCCAGAGGCAACCATCATGCGACAGC-3’

T64A mutation, 5’-CCGCCCCGGCCAGAGACAGCCATCATGCGACAGC-3’

T63A+T64A mutations, 5’-CCGCCCCGGCCAGAGGCAGCCATCATGCGACAGC-3’

T72A mutation, 5’-CGACAGCCACCAGTCGCCCACGCTCCTCAGC-3’

S82A mutation, 5’-CCCCTGCTTCCCGGTGCCGTCAGGCCCCTGC-3’

S117G mutation, 5’-GACTGCGAGGACGGCGGCGATGAGCTAGACT-3’

T164A mutation, 5’-GAAGCCAACTGCCCCGCCAAGCGTCCTGAGG-3’

T174A mutation, 5’-GAAGTGTGCGGGCCCGCACAGTTCCGATGCG-3’

S180A mutation, 5’-CAGTTCCGATGCGTCGCTACCAACATGTGCATCC-3’

T181A mutation, 5’-CAGTTCCGATGCGTCTCTGCCAACATGTGCATCC-3’

S180A+T181A mutations, 5’-CAGTTCCGATGCGTCGCTGCCAACATGTGCATCC-3’

S188G mutation, 5’-ATGTGCATCCCAGCCGGCTTCCACTGTGACG-3’

S195G mutation, 5’-CACTGTGACGAGGAGGGCGACTGTCCTGACC-3’

S201G mutation, 5’-ACTGTCCTGACCGGGGCGACGAGTTTGGCT-3’

Stable expression and cell-based LDL binding assay

Stable cell lines expressing perlecan domain II, I, I+II, mutant domain II and the Fc tag alone were obtained by selection of transfected WT or pgsa-745 mutant CHO cells with neomycin (Invitrogen). The stable cell lines were used for the cell-based LDL binding assay as previously described (1). Equal numbers of cells were split into 4 wells of 6-well plates and

cultured in Ham's F-12 media with 5% lipoprotein deficient serum (a gift from Monty Krieger's lab, MIT) for ~12 h. The cells were washed to remove the proteins in the media, and pre-chilled with serum-free media at 4°C. After 30 min, [I^{125}]-labeled LDL (10 μg/ml) (Biomedical Technologies, Inc.) was added and continued to incubate at 4°C for 1 h. After three washes, the cells were harvested and whole cell lysates were prepared for liquid scintillation counting. The cells from one well chamber were treated at the same condition but without [I^{125}]-labeled LDL and used for cell number counting.

Supplemental figure legends

sFig. 1. **In vitro saturation binding of perlecan domain II with LDL**. The purified sDII-flag (10.0 pM) as in Fig. 2B was bound to anti-flag M2 affinity beads and then incubated with increasing amounts of LDL as indicated for one hr at RT. After wash, the beads were eluted and eluants were analyzed with Western blotting. Quantification analysis was carried out with ImageJ.

sFig. 2. **Functional analysis of perlecan domain II and I in ApoB-100 binding**. **A**. The purified WT DI (Fig. 4A, lane 6) was incubated without (lane 1) or with heparinase I-III (H1-H3, lanes 2-4, respectively) for 24 h. The reaction mixtures and pgsa DI (lane 5) were analyzed as in Fig. 1C. **B**. In vitro binding of the purified proteins with ApoB-100. Top, Equal amounts of the purified proteins as in Fig. 4A were used for in vitro binding assay as Fig. 1E but with human ApoB-100 (5 μg) and 1XTBS. The blot was probed with the anti-ApoB antibody. Bottom, Quantitation of the in vitro binding assay. The average and standard error were based on three independent experiments. **, $p<0.01$ (T-test); n.s., $p>0.05$.

sFig. 3. **Cell-based binding assay for the interaction of perlecan domain II and I with LDL**. **A**. Expression of perlecan domain II, I and I+II in WT CHO cells. Cellular (Cell) and medium

(Med) extracts from the stable cell lines carrying the constructs for Fc, Mu-DII, WT DII, DI, and DI+II as in A were analyzed as in Fig. 1C. The expression of HS is indicated. **B**. Cell-based in vitro binding assay of perlecan domain II and I in LDL binding. Stable cell lines as in A were used for the in vitro binding assay with $[I^{125}]$-labeled human LDL. Total cell lysates were prepared for scintillation counter. Standard deviation was calculated from three independent experiments. *, $p<0.05$ (T-test).

sFig. 4. The multiple-stage mass spectrometry (MS^n) determination of *O*-glycan sialic acid linkage. The upper branch NeuAc linkage of $NeuAcGal_2GlcNAcGalNAc$ (*m/z* 1344) (**A**) and $NeuAc_2Gal_2GlcNAcGalNAc$ (*m/z* 1705) (**B**) was determined (MS^4) by isolating the B/Y lactosamine fragments (*m/z* 472), which are singly-charged sodium adducts. The fragmentation pattern of this disaccharide ion was previously shown to differ depending on NeuAc linkage position (2). In both cases, the fragmentation pattern is consistent with that of a 3-linked NeuAc. For the lower arm NeuAc linkage, where no lactosamine exists, linkage was determined via fragmentation of the lithiated B/Y Gal ion as previously described (3). Collision-induced dissociation of these fragments produces distinctly different fragmentation, depending on location of the hydroxyl to the 3- vs. 6-position. For the sialylated compositions with NeuAc on this arm, including NeuAcGalGalNAc (**C**), $NeuAc_2GalGalNAc$ (**D**), $NeuAcGal_2GlcNAcGalNAc$ (**E**), and $NeuAc_2Gal_2GlcNAcGalNAc$ (**F**), these isolated fragments were all consistent with 3-linked NeuAc. Comparable standards are shown in **G** and **H**, for the 3-linked and 6-linked NeuAc, respectively. NeuAc, neuraminic acid (sialic acid); Gal, galactose.

Supplemental references

1. Ye, Z.J., G.W. Go, R. Singh, W. Liu, A.R. Keramati, and A. Mani. 2012. LRP6 protein regulates low density lipoprotein (LDL) receptor-mediated LDL uptake. *J Biol Chem*. **287**:1335-1344.

2. Ashline, D.J., A.J. Hanneman, H. Zhang, and V.N. Reinhold. 2014. Structural documentation of glycan epitopes: sequential mass spectrometry and spectral matching. *J Am Soc Mass Spectrom*. **25**:444-453.
3. Anthony, R.M., F. Nimmerjahn, D.J. Ashline, V.N. Reinhold, J.C. Paulson, and J.V. Ravetch. 2008. Recapitulation of IVIG anti-inflammatory activity with a recombinant IgG Fc. *Science*. **320**:373-376.

sFigure 1

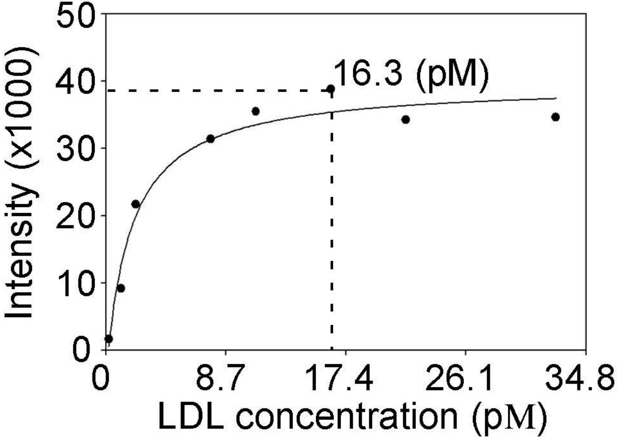

16.3 (pM)
Intensity (x1000)
50
40
30
20
10
0
0
8.7
17.4
26.1
34.8
LDL concentration (pM)

sFigure 2

A

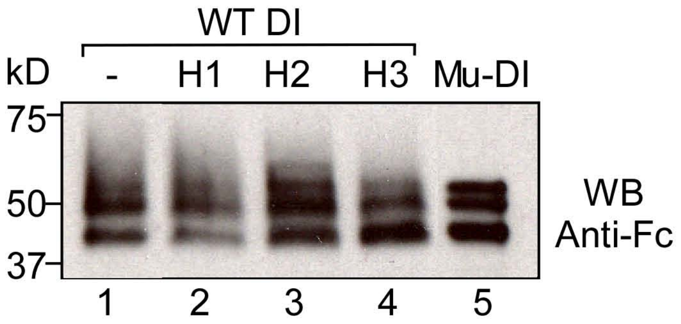
WT DI
kD
-
H1
H2
H3
Mu-DI
75
50
37
WB
Anti-Fc
1
2
3
4
5

B

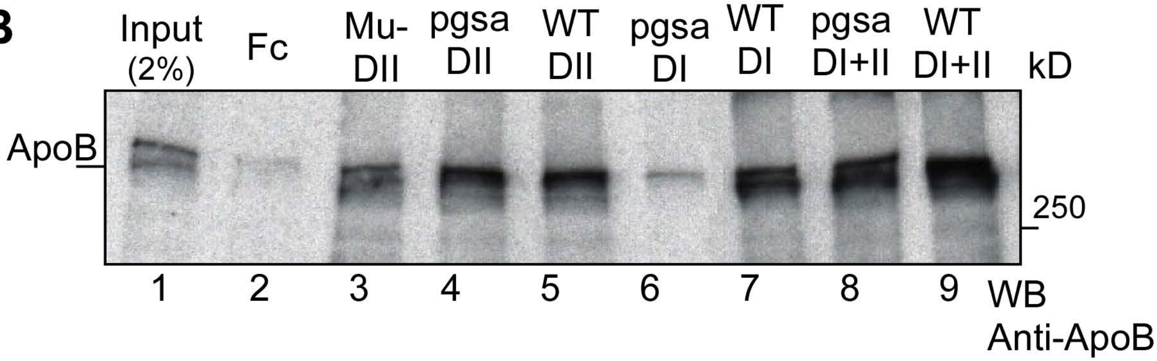
Input
(2%)
Fc
Mu-
DII
pgsa
DII
WT
DII
pgsa
DI
WT
DI
pgsa
DI+II
WT
DI+II
kD
ApoB
250
1
2
3
4
5
6
7
8
9
WB
Anti-ApoB

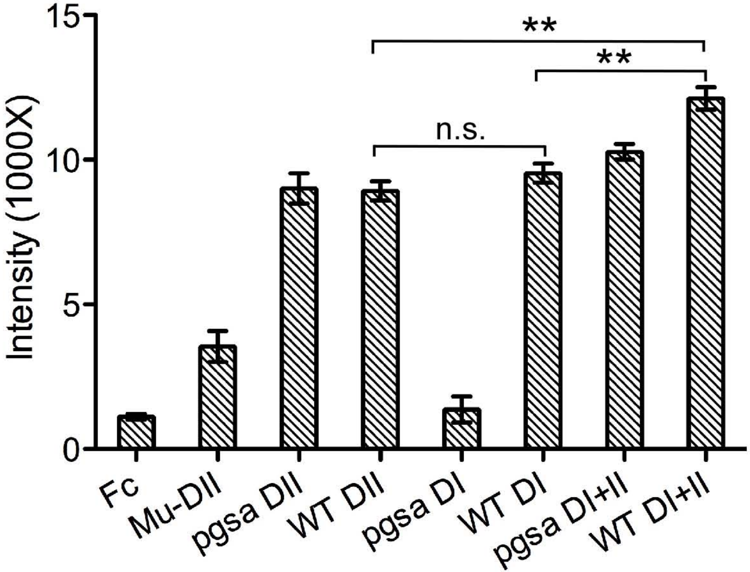
15
10
5
0
Intensity (1000X)
**
**
n.s.
Fc
Mu-DII
pgsa DII
WT DII
pgsa DI
WT DI
pgsa DI+II
WT DI+II

sFigure 3

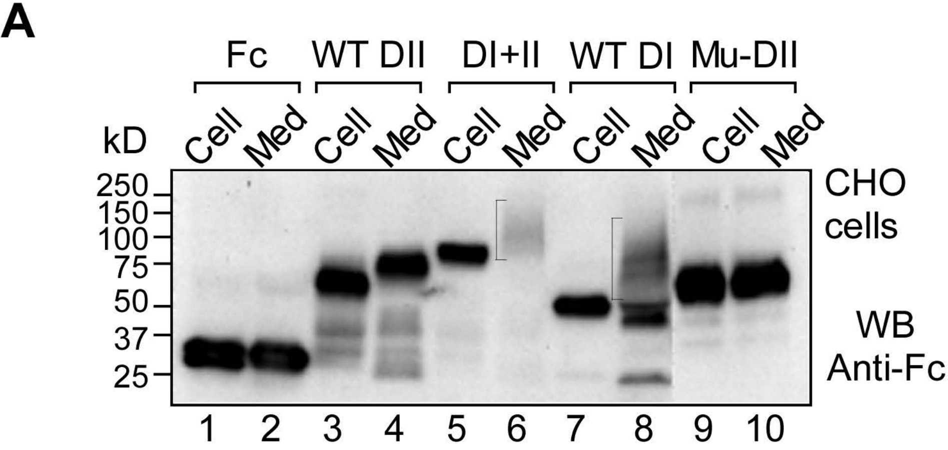
A
Fc
WT DII
DI+II
WT DI
Mu-DII
kD
Cell
Med
Cell
Med
Cell
Med
Cell
Med
Cell
Med
250
150
100
75
50
37
25
CHO cells
WB Anti-Fc
1
2
3
4
5
6
7
8
9
10

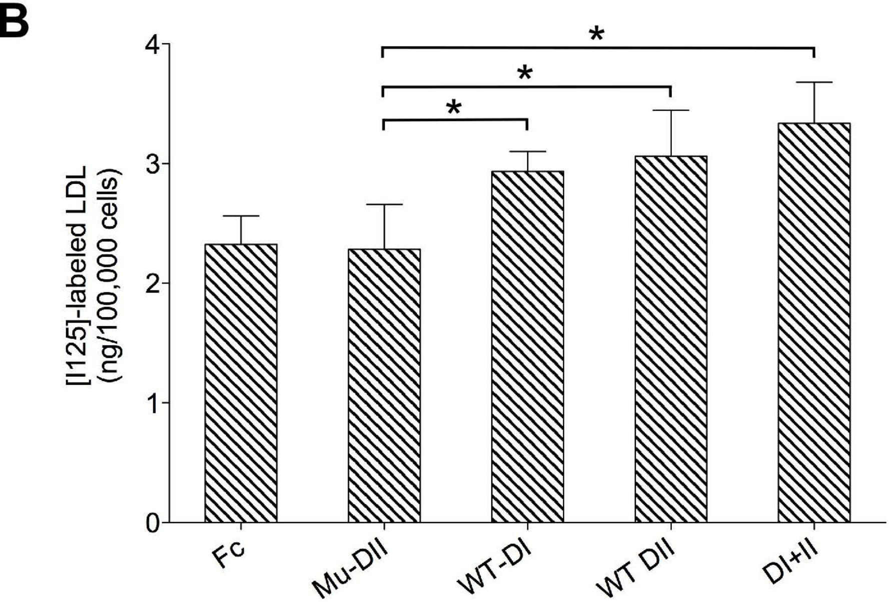
B
[I125]-labeled LDL (ng/100,000 cells)
0
1
2
3
4
*
*
*
Fc
Mu-DII
WT-DI
WT DII
DI+II

sFigure 4

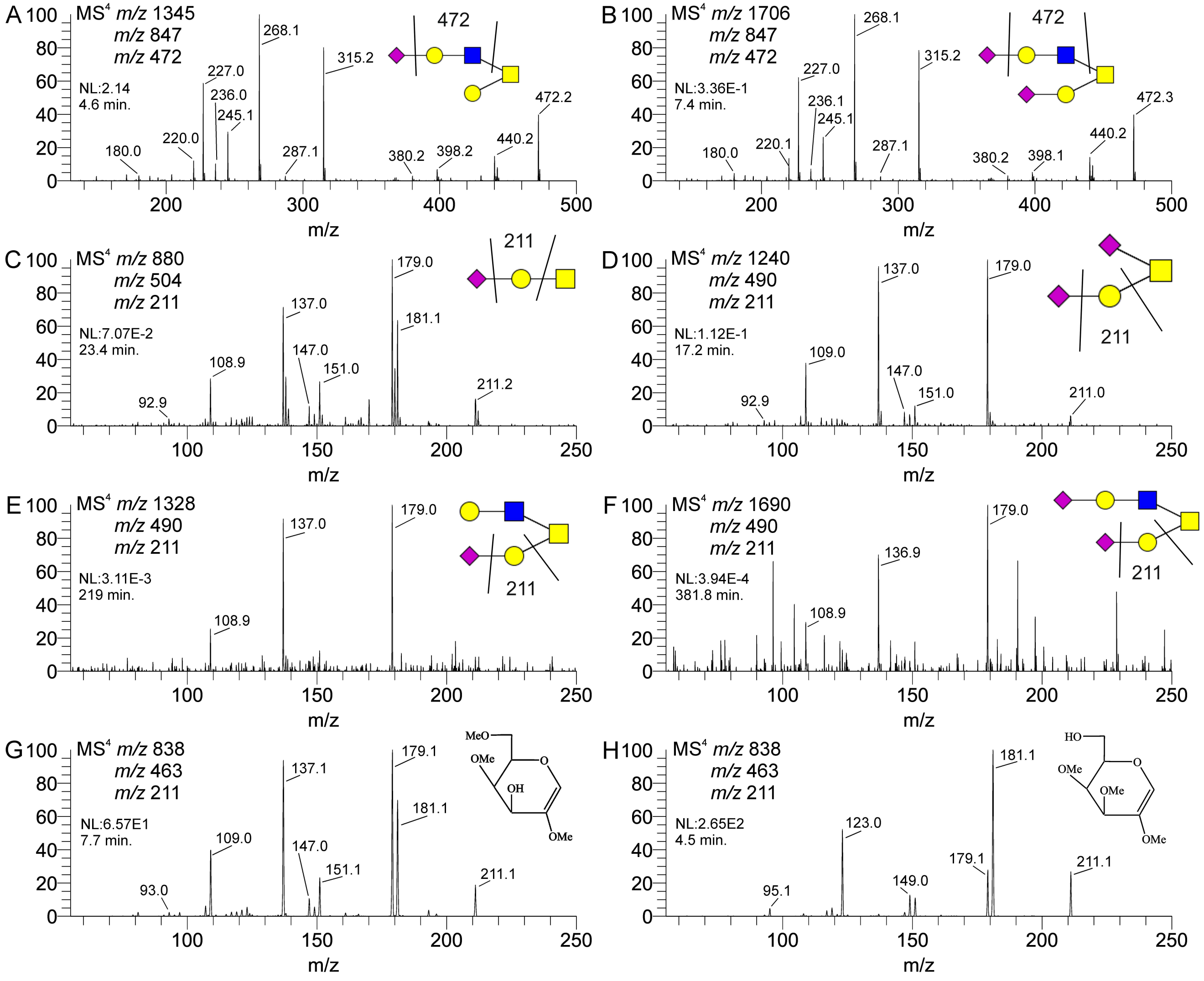

A
MS4 m/z 1345
m/z 847
m/z 472
NL:2.14
4.6 min.
472
268.1
315.2
227.0
236.0
245.1
220.0
180.0
287.1
380.2
398.2
440.2
472.2
B
MS4 m/z 1706
m/z 847
m/z 472
NL:3.36E-1
7.4 min.
472
268.1
315.2
227.0
236.1
245.1
220.1
180.0
287.1
380.2
398.1
440.2
472.3
C
MS4 m/z 880
m/z 504
m/z 211
NL:7.07E-2
23.4 min.
211
179.0
181.1
137.0
147.0
151.0
108.9
92.9
211.2
D
MS4 m/z 1240
m/z 490
m/z 211
NL:1.12E-1
17.2 min.
211
137.0
179.0
109.0
147.0
151.0
92.9
211.0
E
MS4 m/z 1328
m/z 490
m/z 211
NL:3.11E-3
219 min.
211
137.0
179.0
108.9
F
MS4 m/z 1690
m/z 490
m/z 211
NL:3.94E-4
381.8 min.
211
179.0
136.9
108.9
G
MS4 m/z 838
m/z 463
m/z 211
NL:6.57E1
7.7 min.
MeO
OMe
OH
O
OMe
137.1
179.1
181.1
109.0
147.0
151.1
93.0
211.1
H
MS4 m/z 838
m/z 463
m/z 211
NL:2.65E2
4.5 min.
HO
OMe
OMe
O
OMe
181.1
123.0
179.1
149.0
95.1
211.1
m/z
